# CRISPR/Cas9 Targeting of Aldehyde Dehydrogenase 1A1 Reveals Heterogeneous Roles in Radiation Response and Redox Stress Across Clonal Lines in Triple-Negative Breast Cancer

**DOI:** 10.3390/ijms26052303

**Published:** 2025-03-05

**Authors:** Grace O. Ajayi, Aihui Ma, Shirin R. Modarai, Lynn M. Opdenaker, Jennifer Sims-Mourtada

**Affiliations:** 1 Department of Biological Sciences, University of Delaware, 105 The Green, Newark, DE 19716, USA; 2 Cawley Center for Translational Cancer Research, Helen F. Graham Cancer Center and Research Institute, ChristianaCare, 4701 Ogletown Stanton Rd Suite 4300, Newark, DE 19713, USA

**Keywords:** breast cancer, triple negative breast cancer, ALDH1A1, radiation, tumor heterogeneity

## Abstract

The metabolic enzyme aldehyde dehydrogenase 1A1 (ALDH1A1), a cancer stem cell marker associated with poor outcomes in breast cancer, has emerged as a promising therapeutic target in TNBC. The aim of this study was to investigate the role of ALDH1A1 in radiation resistance and redox stress in triple negative breast cancer (TNBC). Functional knockouts of ALDH1A1 were generated by the CRISPR/Cas9-mediated deletion of ALDH1A1 in the SUM159 cell line, and three distinct clonal populations were isolated. Genetic targeting was confirmed by Sanger sequencing, and the loss of ALDH1A1 protein expression was validated by Western blotting. Functional assays assessed ALDEFLUOR activity, cell viability, self-renewal capacity, and reactive oxygen species (ROS) levels with or without radiation in both the bulk population and clonal lines. Interestingly, ALDEFLUOR activity was uniformly lost across all clonal lines; however, functional effects of ALDH1A1 loss on redox stress, survival, and radiation sensitivity were observed in only one clonal population. These findings highlight significant variability in the role of ALDH1A1 among clonal populations, reflecting the complexity of tumor heterogeneity. This underscores the importance of accounting for tumor heterogeneity when targeting ALDH1A1, as certain TNBC subpopulations may rely more heavily on ALDH1A1 function. These insights are critical for developing effective ALDH1A1-targeted therapies.

## 1. Introduction

Triple-negative breast cancer (TNBC) constitutes around 10–15% of total breast cancer cases [1]. It is named for its histological presentation by the lack of favorable staining for estrogen and progesterone receptors, along with the absence of HER2 amplification [2]. These tumors are highly aggressive, displaying substantial genomic instability, and are associated with an unfavorable prognosis and early visceral metastasis [3]. The survival rates for women experiencing relapse within five years of treatment are notably lower compared to those with breast cancer positive for one or two hormone receptors [2,3].

Currently, there are no targeted therapies for triple-negative breast cancer (TNBC). Radiation therapy and chemotherapy have been the main treatment options for locally advanced and metastatic TNBC. However, the effectiveness of radiotherapy or chemotherapeutic strategies has been hindered by inherent or acquired treatment resistance in TNBC [4]. This emphasizes the need for more sensitive and specific therapeutic approaches for this challenging breast cancer subtype [5].

Aldehyde dehydrogenases (ALDHs) are a family of enzymes that catalyze the oxidation of aldehydes to carboxylic acids, playing a crucial role in detoxification, biosynthesis, and various other biological processes [6]. Many ALDH isoforms have been identified in solid tumors underscoring their relevance in tumor progression and therapy resistance [7]. Among the ALDH isoforms present in breast cancer, members of the ALDH1 family, including ALDH1A1 and ALDH1A3, contribute to cancer stemness, progression, and resistance [7,8,9].

ALDH1A1 is particularly significant for its association with treatment resistance and poor outcomes in TNBC [3,10,11,12,13,14]. ALDH1A1 expression in breast cancer is correlated estrogen receptor negativity, high grade, tumor size and stage, lymph node metastasis, and drug resistance [3,12]. ALDH1A1 is best known as a marker of breast cancer stem cells [10], primarily through catalyzing the oxidation of retinaldehyde to retinoic acid—a key regulator of cellular differentiation [8]. ALDH1A1 and ALDH1A3 both drive retinoic acid production in breast cancer, and have been linked to angiogenesis, tumor invasion, and metastasis [8,15]. Additionally, ALDH1A1 plays a direct role in treatment resistance through the metabolism of reactive aldehydes [8,9].

ALDH1A1 contributes to chemoresistance by modulating cellular metabolism and regulating reactive oxygen species (ROS) [9,16,17,18,19,20]. Its expression is upregulated in response to oxidative stress, serving as a protective mechanism to enhance ROS detoxification during aldehyde metabolism and maintain redox balance in both normal and malignant tissues [20,21,22,23]. By detoxifying aldehyde byproducts of lipid peroxidation, such as 4-hydroxynonenal, ALDH1A1 shields cells from oxidative damage induced by chemotherapy [24]. However, ALDH1A1’s role in mitigating these effects enhances chemoresistance, protecting cells under therapeutic stress [8,19,25].

Although ALDH1A1 has been reported to induce radiation resistance in solid tumors, including breast cancer [8,26], the exact mechanism is not clear. Our group has demonstrated that ALDH1A1 activity and expression increase in a dose-dependent manner following ionizing radiation, suggesting a role in managing radiation-induced redox stress [27]. Based on these findings, we hypothesize that targeting ALDH1A1 could disrupt cellular redox balance and sensitize TNBC cells to radiation therapy. To investigate this, we generated genetic knockouts of ALDH1A1 to further explore its role in redox stress and radiation resistance.

## 2. Results

### 2.1. Effect of CRISPR/Cas9 Knockout (KO) of ALDH1A1 SUM159 Bulk Cells

To investigate the role of ALDH1A1 in radiation resistance, we used CRISPR/Cas9 to generate the genetic knockout (KO) of ALDH1A1 in the triple-negative breast cancer (TNBC) cell line SUM159. This cell line was chosen due to its constitutive ALDH1A1 expression and documented ALDH1A1 upregulation following radiation exposure [27]. Three guide RNAs (gRNAs) were designed to target exon 2 of ALDH1A1, which encodes the active domain critical for the ALDH1A1 enzyme’s catalytic function (Figure S1A). Targeting at exon 2 occurs prior to the substrate binding domain [28]. This exon was also selected for targeting as it is common to all ALDH1A1 variants. The commercial gRNAs were predetermined to have low off target effects and induce mutations that result in a frameshift, disrupting the reading frame and impairing the production of a functional protein. These guides were complexed to Cas9 as single guides or in pairs and the ribonuclear complexes (RNP1, RNP2, and RNP3 for single guides or RNP2 + 3 for combination guides) were delivered to SUM159 cells via nucleofection.

**Figure 1:**
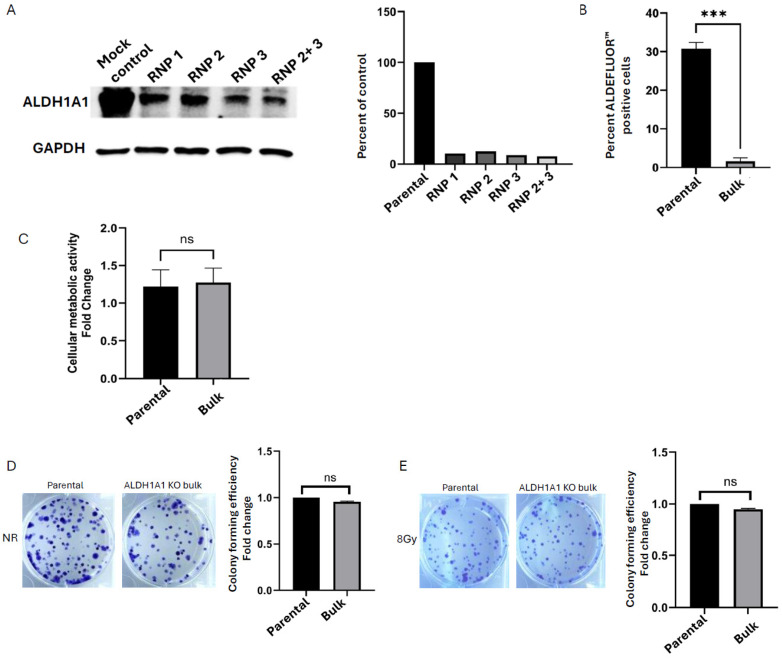
ALDH1A1 knockout bulk cells does not impact cellular metabolism, colony formation, or radiation response. (**A**) Western blot validation and quantification of ALDH1A1 knockout (KO) in targeted CRISPR/Cas9 ribonucleoprotein (RNP) targeted bulk cell populations. Data is represented as percent control of ALDH1A1 protein expression normalized to GAPDH. (**B**) Percent of positive cells as determined by the ALDEFLUOR^TM^ assay in ALDH1A1 KO bulk cells and the parental cell line. (**C**) Alamar blue assay measured cellular metabolism in ALDH1A1 KO bulk cell line. Fold change was expressed as a ratio of day 5/day 1. Bulk KO of ALDH1A1 showed no change in colony formation without (**D**) or with 8 Gy radiation (**E**), as compared to the parental cell line. Data is plotted as the mean ± standard error of the mean normalized to respective control cells. *** *p* < 0.001, ns = not significant.

ALDH1A1 expression level was screened and validated at the protein level (Figure 1A) in the bulk population of transfected cells. We confirmed that using a combination of two different gRNAs to form our ribonucleoprotein complex (RNP2 + 3) was more efficient than the targeting performed with the single gRNAs, and reduced ALDH1A1 expression by 92%. These cells were selected for further study.

Aldehyde dehydrogenases, particularly ALDH1A1 and ALDH1A3, have been shown to drive retinoic acid activity, as measured by the ALDEFLUOR^TM^ assay [8]. To assess the functional impact of ALDH1A1 KO in the bulk SUM159 cells, we tested for the presence of ALDEFLUOR^TM^ high cells in the ALDH1A1 KO bulk cells and found that the loss of ALDH1A1 reduced ALDEFLUOR^TM^ activity significantly compared to the parent cell line (Figure 1B and Figure S1B). We further performed a metabolic assay to determine cell growth and viability. Cells were plated and cellular metabolic activity was measured on day 1 and day 5 using an Alamar Blue assay. No significant difference was observed in bulk ALDH1A1 KO cells compared to control cells (Figure 1C). Additionally, colony formation assays were performed to evaluate reproductive integrity, revealing no significant difference in colony-forming ability between SUM159 parental and ALDH1A1 KO bulk cells (Figure 1D). We also tested colony-forming efficiency in response to 8Gy radiation. This dose was chosen as it was found to have the highest increase in ALDEFLUOR^TM^ activity and ALDH1A1 expression after radiation in our previous study [29]. As shown in Figure 1E, no significant differences in radiation response were observed between the SUM159 control and ALDH1A1 KO bulk cells. These findings indicated that while ALDH1A1 contributes to ALDEFLUOR^TM^ activity, its loss does not significantly impact the overall metabolic activity, clonogenic potential, or radiation response of bulk SUM159 KO cells.

### 2.2. Clonal Cell Lines Derived from Bulk Knockout Cells Have Decreased ALDEFLUOR^TM^ Activity

**Figure 2:**
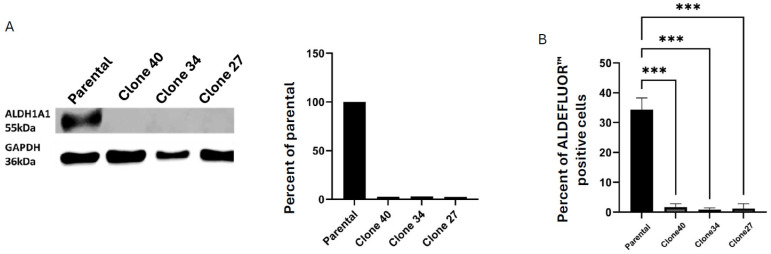
Clonally derived ALDH1A1 knockout cell lines have decreased ALDEFLUOR^TM^ activity. (**A**) Western blot shows the loss of ALDH1A1 protein expression in the clonally derived KO cell lines compared to the SUM159 parental cell line. (**B**) ALDEFLUOR^TM^ assay shows a significant decrease in ALDEFLUOR^TM^ activity in all three clonal lines compared to the parental cell line. Data represent the mean ± SEM normalized to the respective control cells. *** *p* < 0.001.

The bulk population of targeted cells contains a mixture of cells with varying levels of ALDH1A1 knockout. To ensure a complete loss of ALDH1A1 expression, we isolated single-cell clones from the ALDH1A1 KO bulk population. Three clonal lines (clone 40, clone 34, and clone 27) were confirmed to be complete knockouts via Western blotting (Figure 2A) and Sanger sequencing (Figure S2A). Analysis of ALDEFLUOR^TM^ activity in all three clonal lines revealed significant decreases in ALDEFLUOR^TM^ positivity compared to the parental line (Figure 2B and Figure S2B). These results indicate a critical role for ALDH1A1 in ALDEFLUOR^TM^ activity in the three knockout lines.

### 2.3. Clonal Differences in the Effect of ALDH1A1 Knockout on Cellular Metabolic Activity and Colony Formation

**Figure 3:**
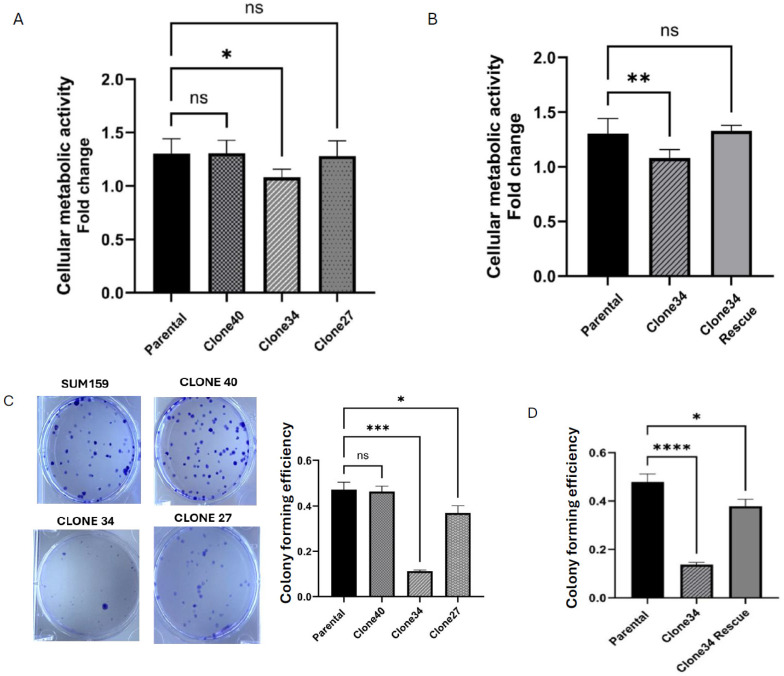
Effect of ALDH1A1 loss on cellular metabolism and survival. (**A**) Alamar blue assay measured cellular metabolism in ALDH1A1 knockout (KO) clonal cell lines. Fold change was expressed as a ratio of day 5/day 1. (**B**) Rescue of ALDH1A1 expression restored cellular metabolism levels in clone 34. (**C**) Colony forming efficiency of parental cells and ALDH1A1 KO cell lines. Representative images of each cell line are shown. (**D**) Rescue of ALDH1A1 expression in clone 34 restored colony forming efficiency. Data represents the mean ± Standard error of the mean normalized to respective parental cells. * *p* < 0.05, ** *p* < 0.01, *** *p* < 0.001, **** *p* < 0.0001, ns = not significant.

To assess the impact of ALDH1A1 KO on cellular metabolic activity in clonal lines, we used the Alamar Blue assay as described previously. Consistent with results in the bulk population, no significant differences in cellular metabolism were observed in Clones 40 and 27. However, Clone 34 exhibited a marked reduction in metabolic activity over time compared to the parental line (Figure 3A). Using a lentiviral vector, we were successfully able to restore ALDH1A1 expression in all KO clones (Figure S3). The rescue of ALDH1A1 expression restored cellular metabolic activity in Clone 34, confirming that the observed metabolic loss was specifically due to the absence of the ALDH1A1 protein (Figure 3B).

Next, we evaluated the effect of ALDH1A1 KO on colony formation in these clonal lines. As shown in Figure 3C, significant reductions in colony forming efficiency were observed in Clones 34 and 27, while Clone 40 showed no notable change. Consistent with the previous experiment, restoration of ALDH1A1 expression rescued colony formation in Clone 34, without affecting cellular metabolic activity (Figure 3D and Figure S4B). These findings demonstrate that ALDH1A1 plays a crucial role in maintaining cellular growth and colony forming ability in certain clonal populations, underscoring clonal variation in dependence on ALDH1A1.

### 2.4. Clonal Differences in the Effect of ALDH1A1 Knockout on Viability, Apoptosis, and Reactive Oxygen Species

**Figure 4:**
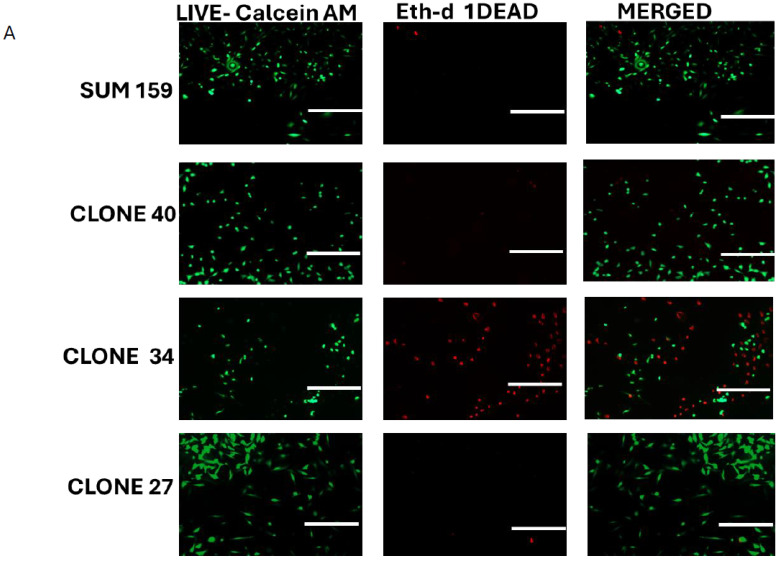

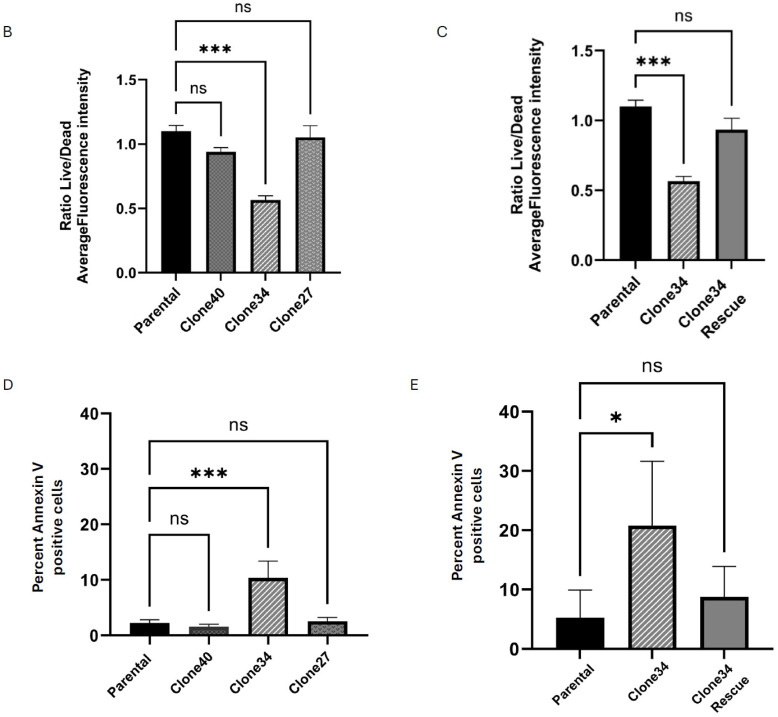
Effect of ALDH1A1 loss on cell viability and survival. (**A**) Representative images of live/dead staining in SUM159 cell, ALDH1A1 knockout (KO) clones, and upon rescue of ALDH1A1 expression in clone 34, magnification = 100X. Scale bar = 200 μm. (**B**) Cell viability determined using Calcein-AM (green) and Ethd-1 (red) and expressed as a ratio of live/dead. (**C**) Rescuing ALDH1A1 expression in clone 34 overcomes loss of viability as detected by Calcein-AM and Ethd-1 fluorescence reading. Data is expressed as a ratio of live/dead. (**D**) Annexin V analysis in ALDH1A1 KO clones showing a significant increase in Annexin V positive cells in clone 34. (**E**) Rescue of ALDH1A1 expression in clone 34 reduces apoptosis measured by Annexin V positive cells. Data is plotted as the mean ± standard error of the mean. * *p* < 0.05, *** *p* < 0.001, ns = not significant.

To investigate the effect of ALDH1A1 KO on cell viability in clonal lines, we performed Calcein-AM/ETHD-1 staining to detect live and dead cells. While no significant changes in viability were observed in Clones 27 and 40, Clone 34 exhibited a significantly lower number of live cells, and a higher number of dead cells compared to the parental SUM159 line (Figure 4A,B). This reduction in viability in Clone 34 was rescued by the overexpression of ALDH1A1 (Figure 4C and Figure S4A).

**Figure 5:**
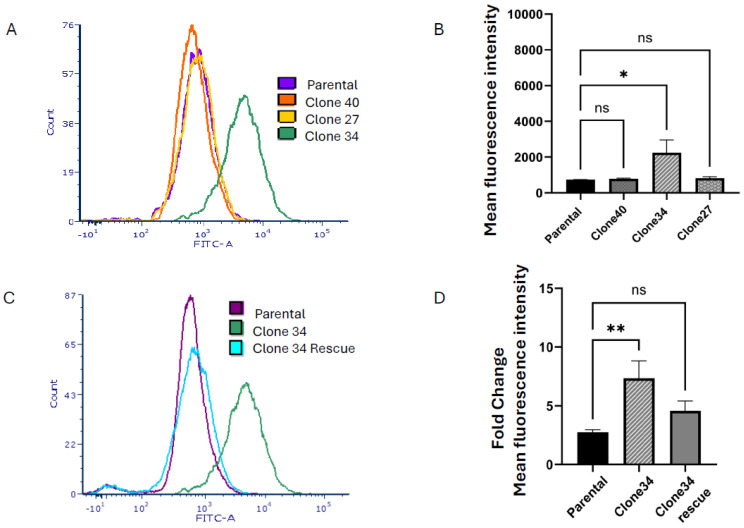
Knockout of ALDH1A1 increases reactive oxygen species. (**A**) Reactive oxygen species (ROS) generation in the different clones as measured by Fluorescein isothiocyanate (FITC-A) intensity shown in a representative histogram. (**B**) Graphical representation of mean fluorescence intensity. (**C**) ROS generation in ALDH1A1 knockout clone 34 and clone 34 rescue as measured by FITC-A intensity as shown in a representative histogram. (**D**) Graphical representation of mean fluorescence intensity represented as fold change of parental. Rescue of ALDH1A1 reduced ROS accumulation in clone 34 represented by fold change compared to the parental. Data is plotted as the mean ± standard error of the mean. * *p* < 0.05, ** *p* < 0.01, ns = not significant.

Furthermore, Annexin-V/PI staining revealed an increased percentage of Annexin V positive cells in Clone 34 compared to the parental line, which was similarly rescued by ALDH1A1 overexpression (Figure 4D,E and Figure S5). Given ALDH1A1’s role in ROS metabolism, we also measured ROS levels in the clonal lines. As shown in Figure 5A,B, ROS levels were significantly elevated in Clone 34 compared to the parental line, and this effect was reversed by rescuing ALDH1A1 expression (Figure 5C,D). These findings suggest that Clone 34 depends on ALDH1A1 for maintaining redox balance and cell viability, unlike other clonal lines.

### 2.5. Radiation Sensitivity in ALDH1A1 Knockout Clones

**Figure 6:**
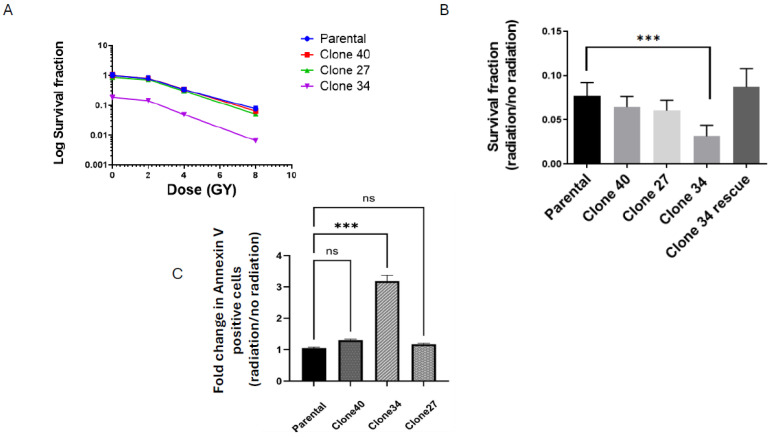
Loss of ALDH1A1 significantly affects colony forming efficiency and increases radiation sensitivity in ALDH1A1 knockout clonally derived cell lines. (**A**) Clonogenic curve shows a decreased surviving fraction in clone 34 only compared to the parental line. (**B**) Surviving fraction at 8 Gy radiation in ALDH1A1 knockout (KO) clones and the parental calculated as radiation over no irradiation for each individual line. Clone 34 shows a significantly decreased survival fraction. Rescue of ALDH1A1 in clone 34 improves survival fraction to that of the parental line. (**C**) Significant increase in apoptosis in clone 34 only after radiation exposure plotted as Annexin V positive cells treated with 8 Gy over that of non-irradiated cells and plotted as fold change over parental. Data is plotted as mean ± Standard error of the mean. *** *p* < 0.001, ns = not significant.

To evaluate the effects of radiation on ALDH1A1 knockout clones, we performed clonogenic assays on the parental SUM159 line and knockout clones at radiation doses of 2, 4, and 8 Gy. Only Clone 34 showed a significant reduction in survival fraction upon radiation exposure (Figure 6A and Figure S6A,B), with the greatest reduction observed at 8 Gy. This effect persisted even after normalization to non-irradiated colony formation (Figure 6B) and was attenuated upon the rescue of ALDH1A1 expression in Clone 34 (Figure 6B and Figure S6B). Clone 27, which exhibited reduced colony formation under non-irradiated conditions, showed no additional sensitization to radiation (Figure 6B).

To further investigate the role of ALDH1A1 in radiation resistance, we assessed radiation-induced apoptosis. Following 8 Gy radiation, apoptosis levels were measured via flow cytometry 3 days post-treatment. Clone 34 exhibited a marked increase in apoptosis compared to other clones and the parental line, an effect that was rescued by ALDH1A1 expression (Figure 6C and Figure S6C). These findings suggest that ALDH1A1 is particularly critical for radiation resistance in Clone 34.

**Figure 7:**
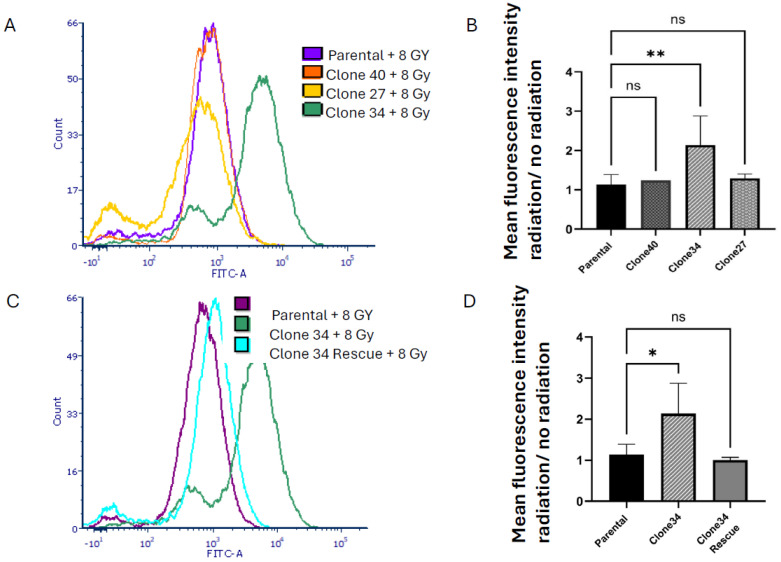
Knockout of ALDH1A1 increases reactive oxygen species levels in radiation response. (**A**) Reactive oxygen species (ROS) generation in the different clones after 8 Gy radiation as measured by Fluorescein isothiocyanate (FITC-A) intensity shown in a representative histogram. (**B**) Graphical representation of mean fluorescence intensity of replicates, presented as radiation over no radiation. (**C**) ROS generation in ALDH1A1 KO clone 34 and rescue after treatment with 8 Gy radiation as measured by their mean FITC-A intensity shown in a representative histogram. (**D**) Graphical representation of the mean fluorescence intensity of replicates, presented as radiation over no radiation. Rescue of ALDH1A1 reduced ROS accumulation in clone 34. Data represents the mean ± SEM. * *p* < 0.05, ** *p* < 0.01, ns = not significant.

Radiation is known to increase ROS levels [30], so we examined ROS accumulation in ALDH1A1 knockout clones after 8 Gy radiation. As shown in Figure 7A,B, ROS levels significantly increased in Clone 34 upon radiation, even when normalized to pre-treatment levels. This effect was reversed by restoring ALDH1A1 expression (Figure 7C,D). No significant changes in radiation-induced ROS levels or sensitivity were observed in other clonal lines (Figure S7A,B).

## 3. Discussion

This study used CRISPR/Cas9-generated functional knockouts of ALDH1A1 in TNBC cells to investigate the role of this ALDH isoform in radiation resistance. We observed that the bulk population of ALDH1A1 KO cells did not exhibit significant changes in radiation response upon ALDH1A1 KO. However, the analysis of clonal lines obtained from the bulk ALDH1A1 KO population revealed distinct variations upon the loss of ALDH1A1. Notably, clone 34 demonstrated a significant reduction in metabolic activity, colony-forming ability, and viability driven by increased ROS accumulation and apoptosis under normal culture conditions. These effects were amplified by radiation with significant increases in ROS and cell death after 8 Gy radiation. The reintroduction of ALDH1A1 expression by viral transfection rescued these effects, confirming that they were specifically related to the loss of ALDH1A1. In contrast, clones 27 and 40 did not exhibit significant changes upon the loss of ALDH1A1, even after radiation, suggesting a heterogeneous dependency on ALDH1A1 among different clonal populations. These findings suggest that ALDH1A1 plays distinct roles in different subpopulations, particularly in relation to cellular metabolism and redox stress.

Interestingly, ALDEFLUOR^TM^ activity, a correlate of retinal metabolism [31,32], was significantly reduced in the bulk knockout population as well as in all three clonal populations upon ALDH1A1 knockdown. This finding indicates that while ALDH1A1 may play a universal role in retinal metabolism across tumor cells, its function in managing redox stress may be context-dependent, varying based on the cellular state or genetic background.

Our finding that the loss of ALDH1A1 significantly reduces ALDEFLUOR^TM^ activity contrasts with a prior study that attributed ALDEFLUOR^TM^ activity primarily to the ALDH1A3 isoform [8]. Notably, this study employed knockdown approaches that left residual ALDH1A1 expression. However, while ALDEFLUOR^TM^ activity is correlated with retinoic acid production, further research is needed to elucidate the roles and potential interactions of ALDH isoforms in retinal metabolism.

Radiation is known to induce cell death by increasing oxidative stress [33]. Our study is the first to demonstrate that the loss of ALDH1A1 leads to enhanced radiation-induced cell death through increased ROS accumulation in a TNBC clonal line. This finding is consistent with studies in prostate cancer which have reported increased ROS in response to radiation upon the inhibition of ALDH family activity with the competitive inhibitor DEAB [34]. Likewise, a previous study reported increased radiation-induced death in bulk SUM159 cells upon the partial loss of ALDH1A1 siRNA knockdown. This study utilized fractionated radiation, resulting in significantly higher cumulative doses compared to the single-dose approach in our study [8]. While we did not see a radiation effect in all clonal lines, it is possible that compensatory mechanisms for ALDH1A1 loss exist in some populations at lower radiation doses but may be lost at higher cumulative doses observed with fractionated radiation.

Our group and others have shown that ALDH1-positive cells are resistant to radiation in TNBC and that ALDEFLUOR^TM^ activity is upregulated in both ALDH-positive and negative cells post-irradiation [27,35]. We previously observed a dose-dependent upregulation of ALDEFLUOR^TM^ activity correlating with the increased expression of both ALDH1A1 and ALDH1A3 [27]. In prostate cancer, knockdown of both ALDH1A1 and ALDH1A3 enhanced radio sensitization, but only ALDH1A1 knockdown impaired DNA repair, suggesting a unique role for ALDH1A1 in maintaining cellular homeostasis upon radiation [26]. Although our findings are consistent with this study, we did not investigate the role of ALDH1A3, and it remains unclear if ALDH1A3 can compensate for ALDH1A1 loss in TNBC.

Our study indicates that ALDH1A1 primarily promotes radiation resistance in a distinct subpopulation of cells. We observed that one clonal line relies on ALDH1A1 activity, both under normal conditions and following radiation exposure, as evidenced by reduced colony formation, increased ROS accumulation, and heightened apoptosis in the absence of ALDH1A1. Cancer stem-like cells have been reported to have lower ROS [36], and previous research has established that ALDH1A1 is highly expressed in cancer stem-like cells and plays a critical role in the mitigation of redox stress in these cells [19,37,38,39,40]. We have previously shown that cancer stem cells are more sensitive to the inhibition of STAT3-driven ALDH activity upon radiation than their non-stem cell components [27]. However, the phenotypes of our clonal populations remain uncharacterized. Studies are underway to profile these clones and to expand functional knockout studies to include other TNBC cell lines and patient-derived samples to understand ALDH1A1 function across different tumor microenvironments and genetic backgrounds.

This study was conducted using the SUM159 cell line, derived from the primary tumor of a metastatic anaplastic breast cancer. SUM159 represents a claudin-low TNBC phenotype [41], a subtype characterized by cancer stem cell-like features and low genomic stability [41,42], underscoring its heterogeneity. Although SUM159 has been reported to be an intermediate tumorigenic cell line with limited metastatic potential [43], it has tumorigenic variants [44,45], highlighting its complex and heterogeneous nature. Previous studies have shown that SUM159 exhibits ALDH1A1 expression and function similar to other TNBC cell lines [8]. We and others have shown that ALDH1A1 is upregulated in response to radiation in SUM159 as well as other claudin low breast cancer cell lines [27,35]. Notably, SUM159 is among the most radioresistant breast cancer cell lines [46]. It remains to be determined whether the clones isolated in our study represent radioresistant or metastatic components of SUM159. Moreover, whether other claudin low TNBC cell lines or other breast cancer subtypes display similar heterogeneity in ALDH1A1 function remains to be determined. Studies are underway to profile these clones and to expand functional knockout studies to include other TNBC cell lines and patient-derived samples to understand ALDH1A1 function across different tumor microenvironments and genetic backgrounds. 

CRISPR/Cas9-mediated gene editing can introduce off-target effects which may influence experimental outcomes [47]. To mitigate this, we used commercially available sgRNAs which were predicted to have limited off target interactions. Additionally, we rescued the ALDH1A1 KO by overexpressing the full-length ALDH1A1 protein, confirming that the observed phenotypes were specifically due to ALDH1A1 loss rather than off-target effects. To further validate our results, future studies should incorporate sgRNAs targeting different exons to ensure that the observed phenotypes are not due to exon-specific effects and to confirm that ALDH1A1 loss, rather than off-target modifications, drives the observed changes.

Although our findings highlight the role of ALDH1A1 in radiation resistance, the underlying mechanism remains unknown. Further mechanistic studies should focus on identifying differential pathways and alternative enzymes, such as ALDH1A3, that may compensate for the loss of ALDH1A1 and contribute to radiation protection. Additionally, these observations need to be validated in in vivo models to better understand the impact of ALDH1A1 loss within the tumor microenvironment and its potential therapeutic implications.

## 4. Materials and Methods

### 4.1. Cell Culturing

SUM159 cells were obtained from Asterand BioIVT (Westbury, NY, USA). SUM159 cells were cultured in Ham’s F12 medium (ThermoFisher Scientific, Carslbad, CA, USA) and supplemented with 5% FBS, 1× antibiotic/antimycotic, 2 μg/mL insulin (ThermoFisher Scientific), and 0.1 mg/ml hydrocortisone (Sigma Aldrich, St. Louis, MO, USA). 

### 4.2. CRISPR/Cas9 Targeting

To generate ALDH1A1 knockout cell lines, we chose three single guide RNAs (sgRNAs 2, 3, and 4) from the Synthego website (Redwood City, CA, USA) targeting exon 2 of the ALDH1A1 gene, each containing the requisite NGG PAM sequence. The sgRNAs with the lowest off-target edits and highest editing efficiency at the target site were chosen. These guide RNAs were then complexed with Cas9 endonuclease to form RNP complexes. SUM159 cells were cultured under standard conditions until 70–80% confluence, after which they were subjected to nucleofection with the RNP complexes to facilitate nuclear entry using the Lonza 4D nucleofector. Following a 72 h recovery period, the initial validation of ALDH1A1 KO efficiency was performed via Western blot analysis. The most effective guide, as determined by protein knockdown levels, was selected for subsequent experiments. The bulk population of targeted cells was then sorted into 96-well plates using fluorescence-activated cell sorting (FACS). Individual clones were screened and expanded under standard culture conditions, followed by the comprehensive validation of ALDH1A1 knockout at the DNA and protein levels. KO efficiency was validated using sanger sequencing results to analyze in Synthego’s ICE software version 3, and target protein expression level was validated by immunoblotting. We selected 2–3 clones exhibiting complete ALDH1A1 knockout for further experimentation. These validated knockout clones were then preserved for future use. Throughout the process, appropriate controls were maintained, and all experimental parameters were thoroughly documented to ensure reproducibility.

### 4.3. Western Blot Analysis

SUM159 cells and the clonally derived cell lines were treated and incubated as indicated. When cells reached 70–80% confluency, they were harvested in RIPA buffer (Sigma Aldrich) with phosphatase and protease inhibitors EDTA-free (ThermoFisher Scientific). BCA assays were performed to determine protein concentration using Pierce BCA protein assay kit (ThermoFisher). Western blot was performed using 10% ten-well comb precast TGX gel from Bio-rad, Hercules, CA, USA. Gels were transferred to nitrocellulose membranes (LI-COR Biosciences, Lincoln, NE, USA) and blocked in 3% BSA in TBS-T and blotted for ALDH1A1 primary antibody at room temperature (RT). The following antibodies were used. ALDH1A1 Rabbit monoclonal antibody (Cell Signaling Technology, Denvers, MA, USA) at room temperature for 1 hour at a 1:1000 dilution. An anti-rabbit secondary (Jackson Immunoresearch Laboratories, West Grove, PA, USA) was used prior to detecting signal with SuperSignal^TM^ West Dura Extended Duration Substrate (ThermoFisher Scientific). Images were acquired and densitometry was performed using a LI-COR Odyssey imager.

### 4.4. Sanger Sequencing

DNA was extracted from SUM159 parent cells and the clonally derived cell lines using DNeasy blood and tissue kit (Qiagen, Germantown, MD, USA). DNA concentrations were checked using a Nanodrop one (ThermoFisher Scientific) and DNA was diluted to 25 ng/µL or less. Primers were obtained from Integrated DNA technologies and then a gradient PCR was run to obtain the optimum annealing temperature of the primers. After this, amplicon PCR was performed and the PCR clean up with the QIAquick PCR purification kit (250) (Qiagen) was performed according to the recommended protocol by the company. Samples were then sent to Azenta, South Plainfield, NJ, USA for Sanger sequencing. Sanger sequencing data were analyzed using ICE software v3 and Benchling (San Francisco, CA, USA).

### 4.5. ALDEFLUOR Assay

ALDEFLUOR assay (Stem Cell Technologies, Vancouver, BC, Canada) was performed according to the manufacturer’s protocol as described in [27]. DEAB gates were set to 0.2%. Assay was performed in three biological replicates.

### 4.6. Colony Forming Assay

Colony forming assays were performed as previously described [27]. SUM159 parent and clonally derived cells were plated into a 100 mm dish at a density of one million cells. Plates were then treated with 2, 4, or 8 Gy. Controls cells were plated similarly but received no radiation. After radiation treatment, cells were split and seeded at low densities from 100–5000 cells in 6 well plates, cultured for 7 days, fixed, stained with crystal violet, and colonies were counted. Experiments were performed at least three times in triplicates. Colony forming efficiency was determined as the number of colonies counted over the number of cells plated. Survival fractions for dose curves were determined as the number of colonies counted for a clone divided by the number of cells plated multiplied by colony forming efficiency of the parental line. Survival fraction for radiation divided by no radiation was determined as the number of colonies counted for a clone divided by the number of cells plated multiplied by the colony forming efficiency of that individual clone.

### 4.7. Apoptosis Assay

Cells were plated at a low density of 150,000 cells per well in a 6-well plate. Cells were left for 72 h and then trypsinized, washed in 1X PBS, and incubated with 1X annexin-binding buffer, Alexa fluor 488 Annexin V component, and propidium iodide solution according to the manufacturer’s protocol (ThermoFisher Scientific). Results were collected by flow cytometry. For radiation response, one million cells were plated in a 100 mm dish and allowed to attach and treated with 8 Gy radiation. The cells were then replated at 15,000 cells per well and the assay was performed as described above.

### 4.8. Live/Dead Assay

The Invitrogen Live/Dead viability/cytotoxicity kit was used according to the manufacturer’s instructions, including Calcein AM visibility dye and EthD-1 dye. Cells were plated at a density of 500 cells per well in a 96-well black clear bottom plate and allowed to grow for 5 days, it was stained with Calcien AM and Ethidium homodimer-1 and read on a plate reader (Tecan, Mannedorf, Switzerland) at their respective fluorescence wavelengths. Alternatively, 1000 cells were used on cover slips and stained. Images were captured using a Zeiss Axioscope microscope (Zeiss, Jena, Germany).

### 4.9. Reactive Oxygen Species

The 2′,7′-dichlorofluorescein diacetate (DCFDA) (Abcam, Cambridge, MA, USA) was used to detect reactive oxygen species (ROS). Cells were plated at a density of 250,000 and allowed to attach. The next day, we incubated 10 µm of DCFDA for 30 min and 100 µm of TBHP (tert-butyl hyperperoxide) as the positive control. The ROS levels were then measured by flow cytometry.

### 4.10. Alamar Blue Viability Assay

A total of 1000 cells were plated in 96 well plates and incubated with Alamar blue (ThermoFisher Scientific) per the manufacturer’s protocol. This was read at the Nanoquant Infinite Pro plate reader (Tecan, Mannedorf, Switzerland) on days one and five, at an absorbance of 570 nm.

### 4.11. Lentiviral Transduction

Lentiviral overexpression of ALDH1A1 was used to rescue ALDH1A1 in the knockout cell lines. The lentiviral construct (Genecopoeia, Rockville, MD, USA) was made by the ALDH1A1 gene under the control of a CMV promoter. This construct also includes Avi and flag tags for protein detection, an SV40 element, and a puromycin resistance gene for selection. The cells of each cell line were seeded and incubated with the lentivirus for 72 h to allow for viral entry and integration into the host genome. Successfully transduced cells were selected using puromycin (ThermoFisher scientific) for 1 week at 10 µg/µL. The overexpression of ALDH1A1 was verified by Western blotting.

### 4.12. Statistical Analysis

Statistical comparisons between two groups (e.g., parent and bulk) were analyzed using Welch’s *t*-test. For all other analyses involving multiple groups, one-way ANOVA was performed with subsequent Dunnett’s post-hoc test. All statistical analyses were conducted using GraphPad Prism version 10.3.0 (507) (GraphPad Software, Boston, MA, USA).

## 5. Conclusions

In conclusion, our study shows the diversity of ALDH1A1’s role in redox balance and resistance to radiation-induced stress among TNBC clonal populations. While certain cellular phenotypes require ALDH1A1 to overcome reactive oxygen species, others do not. The variable responses among TNBC subpopulations to the loss of ALDH1A1 highlight the complexity of its role in TNBC and have implications for the development of ALDH1A1 drug-targeting strategies.

## Data Availability

The original contributions presented in this study are included in the article/Supplementary Material. Further inquiries can be directed to the corresponding author(s).

## Supplementary Materials

The following supporting information can be downloaded at: https://www.mdpi.com/article/10.3390/ijms26052303/s1.
