# Supplementary material for: CRISPR/Cas9 Targeting of Aldehyde Dehydrogenase 1A1 Reveals Heterogeneous Roles in Radiation Response and Redox Stress Across Clonal Lines in Triple-Negative Breast Cancer
Source: Int J Mol Sci. 2025 Mar 5;26(5):2303. doi: 10.3390/ijms26052303 (PMC11900224; doi:10.3390/ijms26052303)
Supplement: Supplementary file 1 [file ijms-26-02303-s001.zip › ijms-3475130-supplementary.pdf]

A

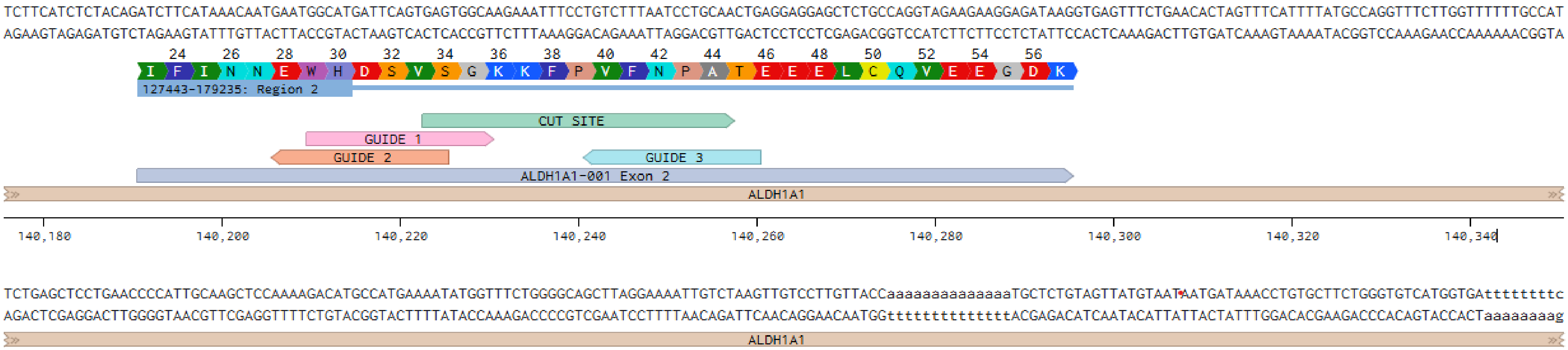
127443-179235: Region 2
CUT SITE
GUIDE 1
GUIDE 2
GUIDE 3
ALDH1A1-001 Exon 2
ALDH1A1
140,180
140,200
140,220
140,240
140,260
140,280
140,300
140,320
140,340
ALDH1A1

B

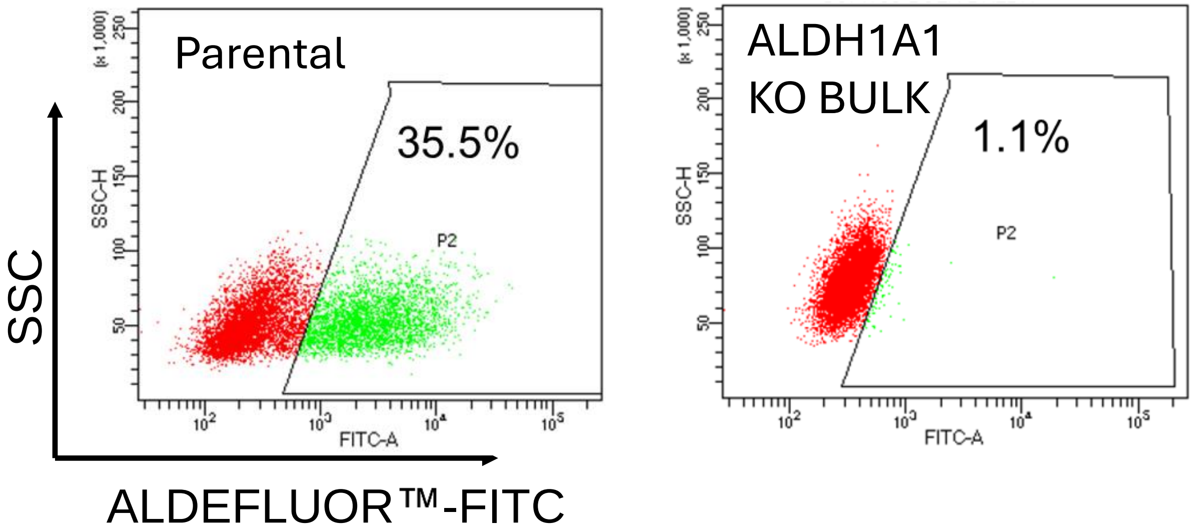
Parental
35.5%
P2
ALDH1A1
KO BULK
1.1%
P2
SSC-H
FITC-A
SSC
ALDEFLUOR™-FITC

Figure S1. Validation of ALHD1A1 knockout and functional effects in bulk cells. (A) Illustration depicting the targeted sequence within exon 2 of ALDH1A1 and the position of each single guide RNA (sgRNA). **(B)** Representative flow cytometry image showing significantly lower ALDEFLUOR™- Fluorescein isothiocyanate (FITC) positive cells in the ALDH1A1 knockout bulk cell line, as compared to the parental SUM159 cells.

A

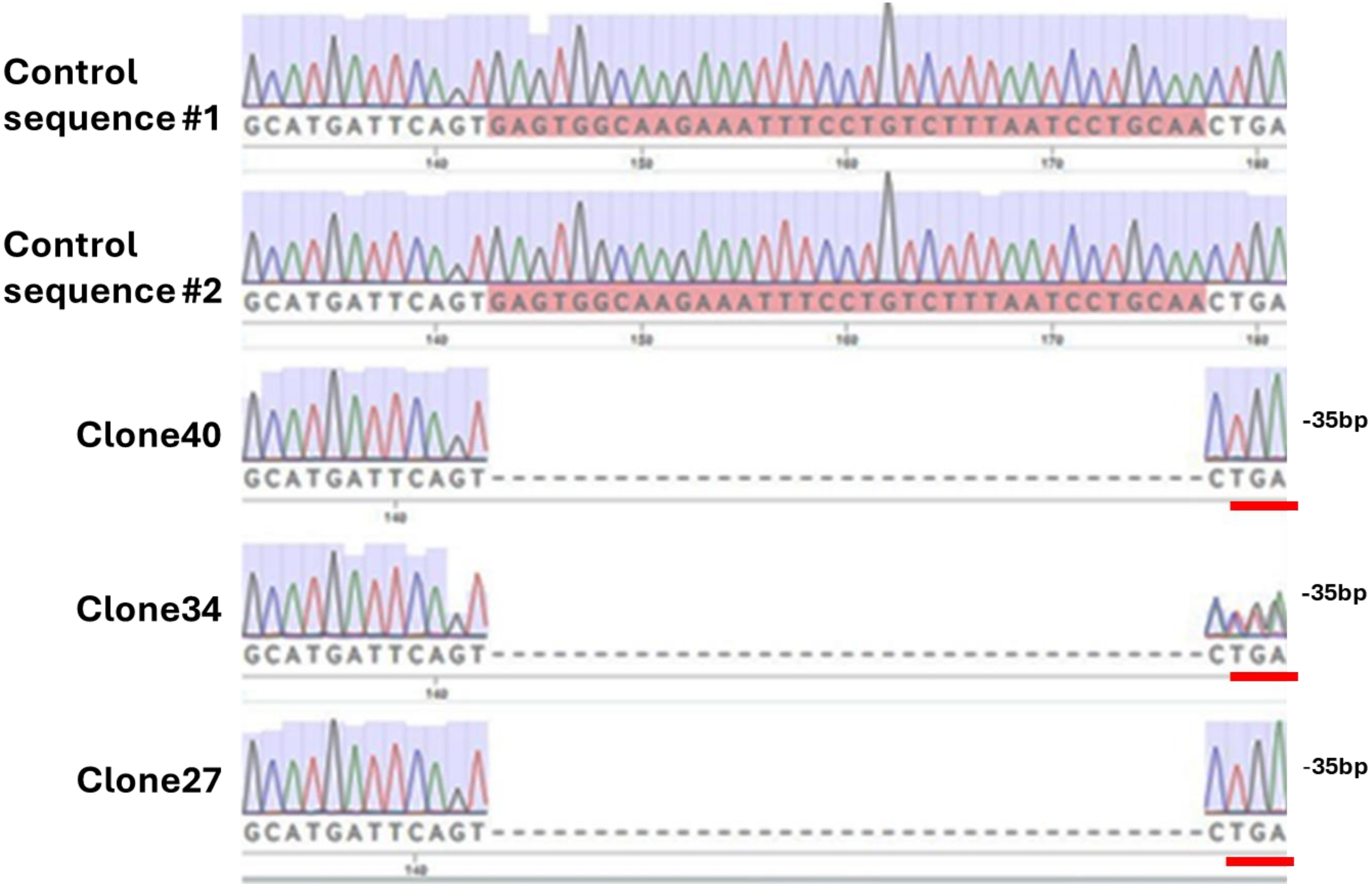

Control sequence #1
GCATGATTCAGTGAGTGGCAAGAAATTTCCTGTCTTTAATCCTGCAACTGA
Control sequence #2
GCATGATTCAGTGAGTGGCAAGAAATTTCCTGTCTTTAATCCTGCAACTGA
Clone40
GCATGATTCAGT-----------------------------------CTGA
-35bp
Clone34
GCATGATTCAGT-----------------------------------CTGA
-35bp
Clone27
GCATGATTCAGT-----------------------------------CTGA
-35bp

B

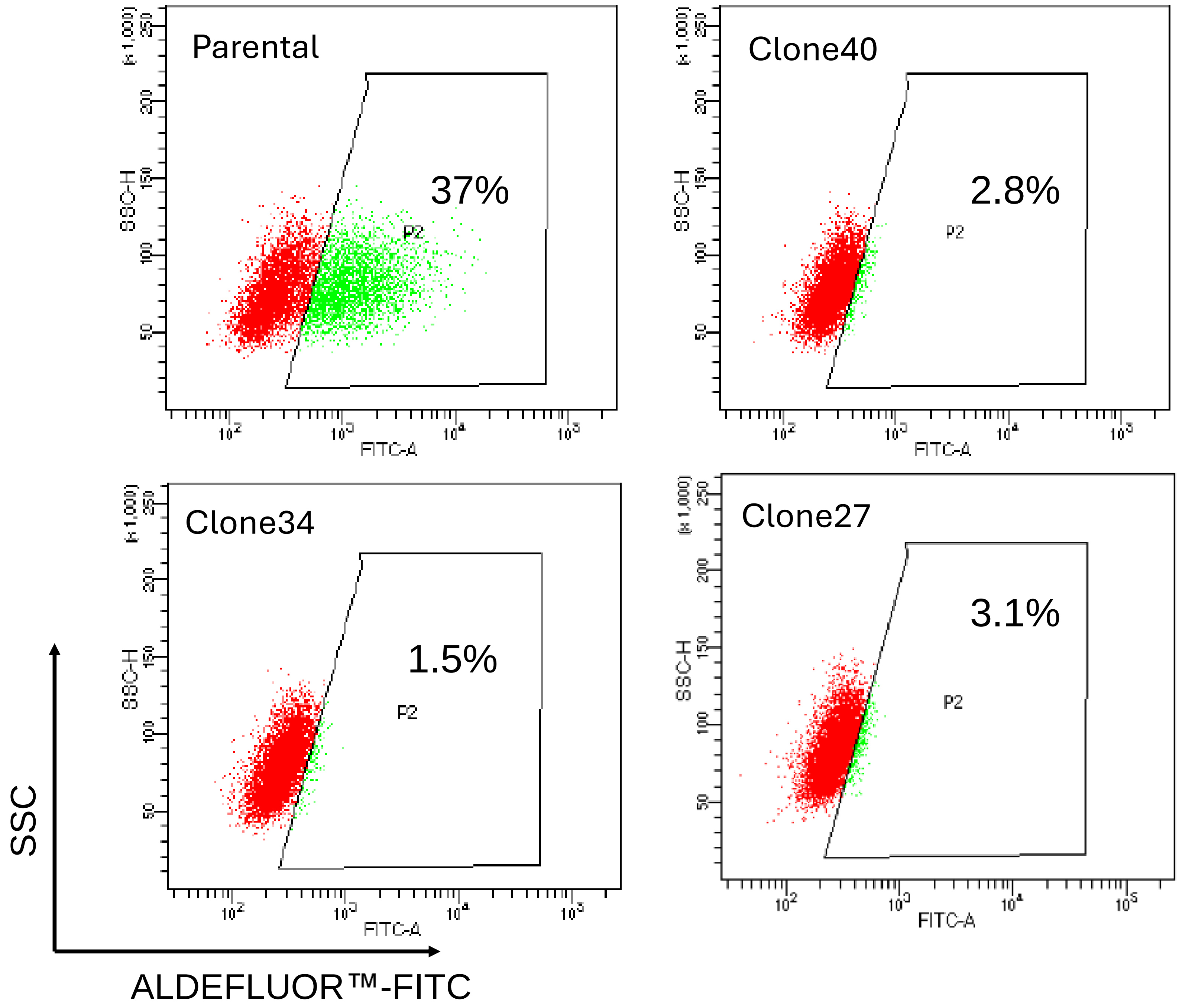
Parental
37%
P2
SSC-H
FITC-A
Clone40
2.8%
P2
SSC-H
FITC-A
Clone34
1.5%
P2
SSC-H
FITC-A
Clone27
3.1%
P2
SSC-H
FITC-A
SSC
ALDEFLUOR™-FITC

Figure S2. Clonal lines of ALDH1A1 KO cells show reduced ALDEFLUOR™ activity compared to parental cells (A) Results of Sanger sequencing showing the SUM159 parental cells, mock control cells, and ALDH1A1 CRISPR/Cas9 RNP targeted clones. A 35 bp deletion is observed in all clones and results in an in frame stop codon (red underline). **(B)** A representative flow cytometry image showing that ALDEFLUOR™-positive cells were significantly lower in the ALDH1A1 KO cell lines (clone 40, clone 34, and clone 27) compared to the parental cells.

ALDH = Aldehyde dehydrogenase, bp = basepair, FITC = Fluorescein isothiocyanate, KO = knockout, SSC= side scatter

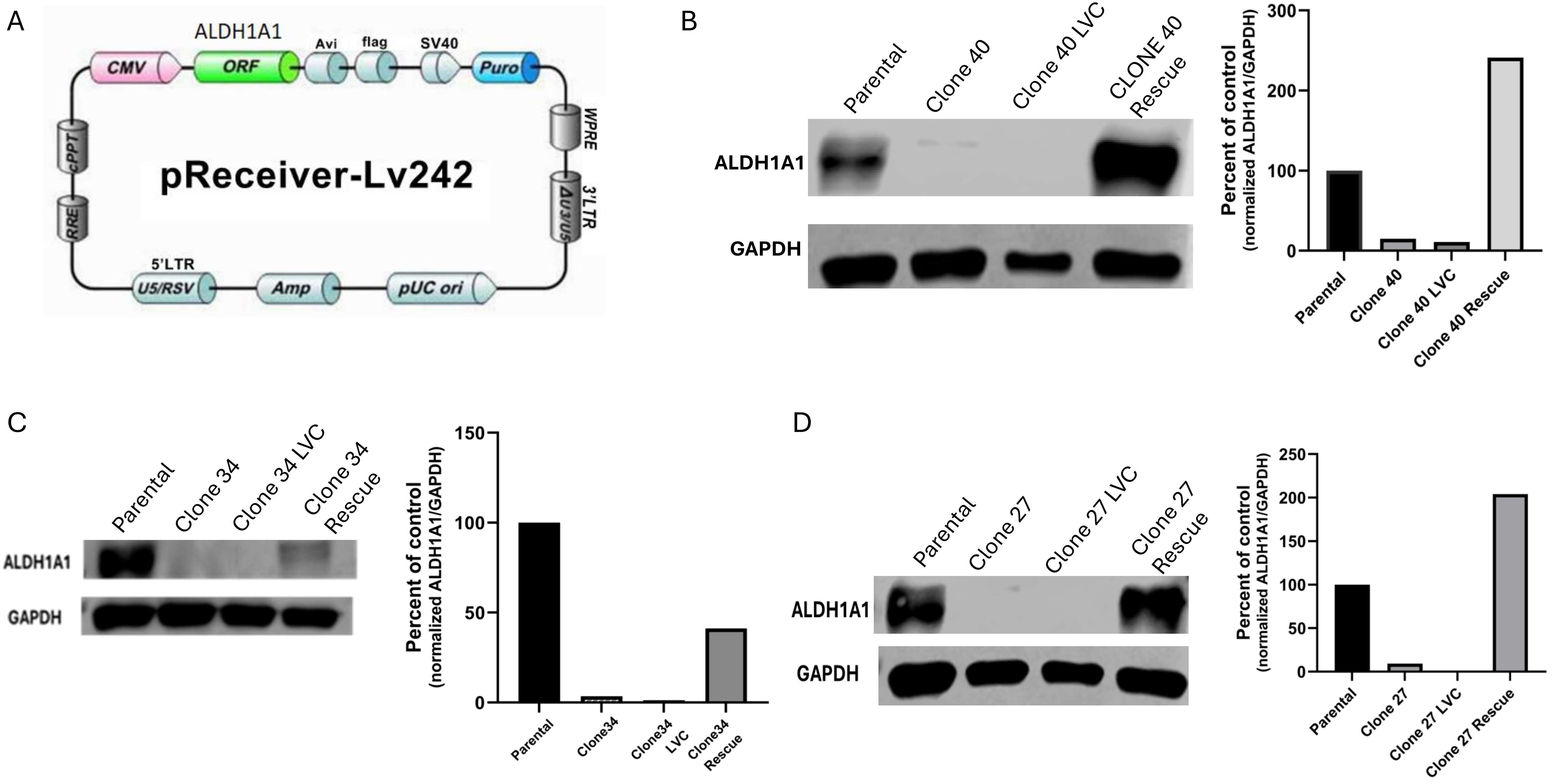
A
ALDH1A1
CMV
ORF
Avi
flag
SV40
Puro
WPRE
3'LTR
ΔU3/U5
cPPT
RRE
pReceiver-Lv242
5'LTR
U5/RSV
Amp
pUC ori
B
Parental
Clone 40
Clone 40 LVC
CLONE 40 Rescue
ALDH1A1
GAPDH
Percent of control (normalized ALDH1A1/GAPDH)
300
200
100
0
Parental
Clone 40
Clone 40 LVC
Clone 40 Rescue
C
Parental
Clone 34
Clone 34 LVC
Clone 34 Rescue
ALDH1A1
GAPDH
Percent of control (normalized ALDH1A1/GAPDH)
150
100
50
0
Parental
Clone34
Clone34 LVC
Clone34 Rescue
D
Parental
Clone 27
Clone 27 LVC
Clone 27 Rescue
ALDH1A1
GAPDH
Percent of control (normalized ALDH1A1/GAPDH)
250
200
150
100
50
0
Parental
Clone 27
Clone 27 LVC
Clone 27 Rescue

Figure S3. Rescue of ALDH1A1 in ALDH1A1 knockout cell lines. (A) Lentiviral transfection plasmid construct for restoration of ALDH1A1 in the ALDH1A1 knockout clonal cell lines. **(B-D)** Western blot confirming the rescue of ALDH1A1 in clonally derived cell lines. Graphs represent densitometry analysis of the blot for clone 40, clone 34 and clone 27. Lentiviral control (LVC) is the same as the knockout cells with the lentivirus with respect to ALDH1A1 protein expression.

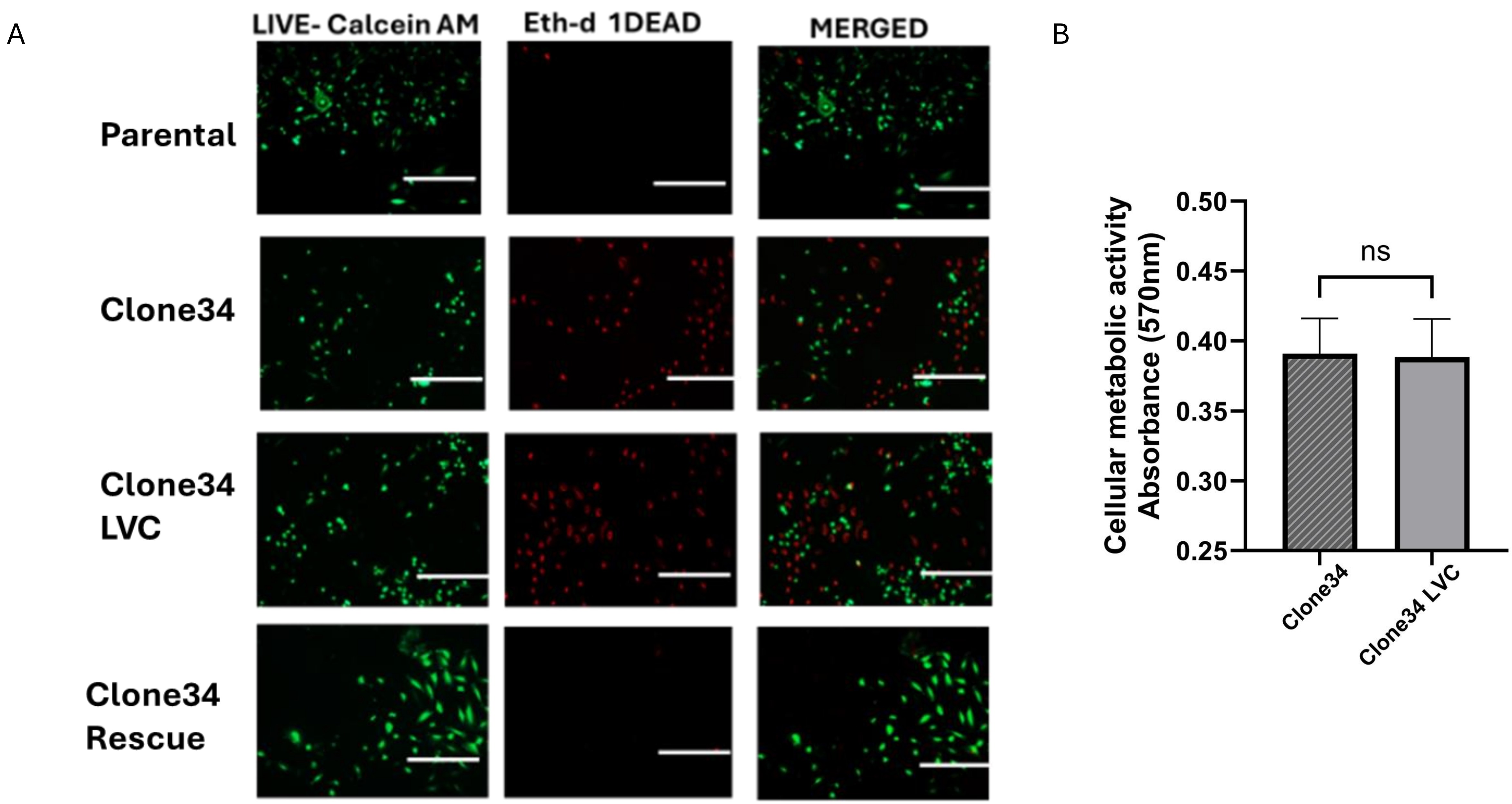
A
LIVE- Calcein AM
Eth-d 1DEAD
MERGED
Parental
Clone34
Clone34 LVC
Clone34 Rescue
B
Cellular metabolic activity Absorbance (570nm)
0.50
0.45
0.40
0.35
0.30
0.25
ns
Clone34
Clone34 LVC

Figure S4. Restoration of live/dead cellular levels and cell metabolism with ALDH1A1 protein rescue in clone 34. (A) Representative images show live/dead staining of clone 34 after rescue of ALDH1A1 protein; magnification 100X. Scale bar = 200 μm **(B)** Alamar Blue cell viability assay shows that clone 34 knockout cells and clone 34 lentiviral control levels are the same. Data represents the mean ± SEM normalized to respective parental cells. ns = not significant.

LVC = lentiviral control

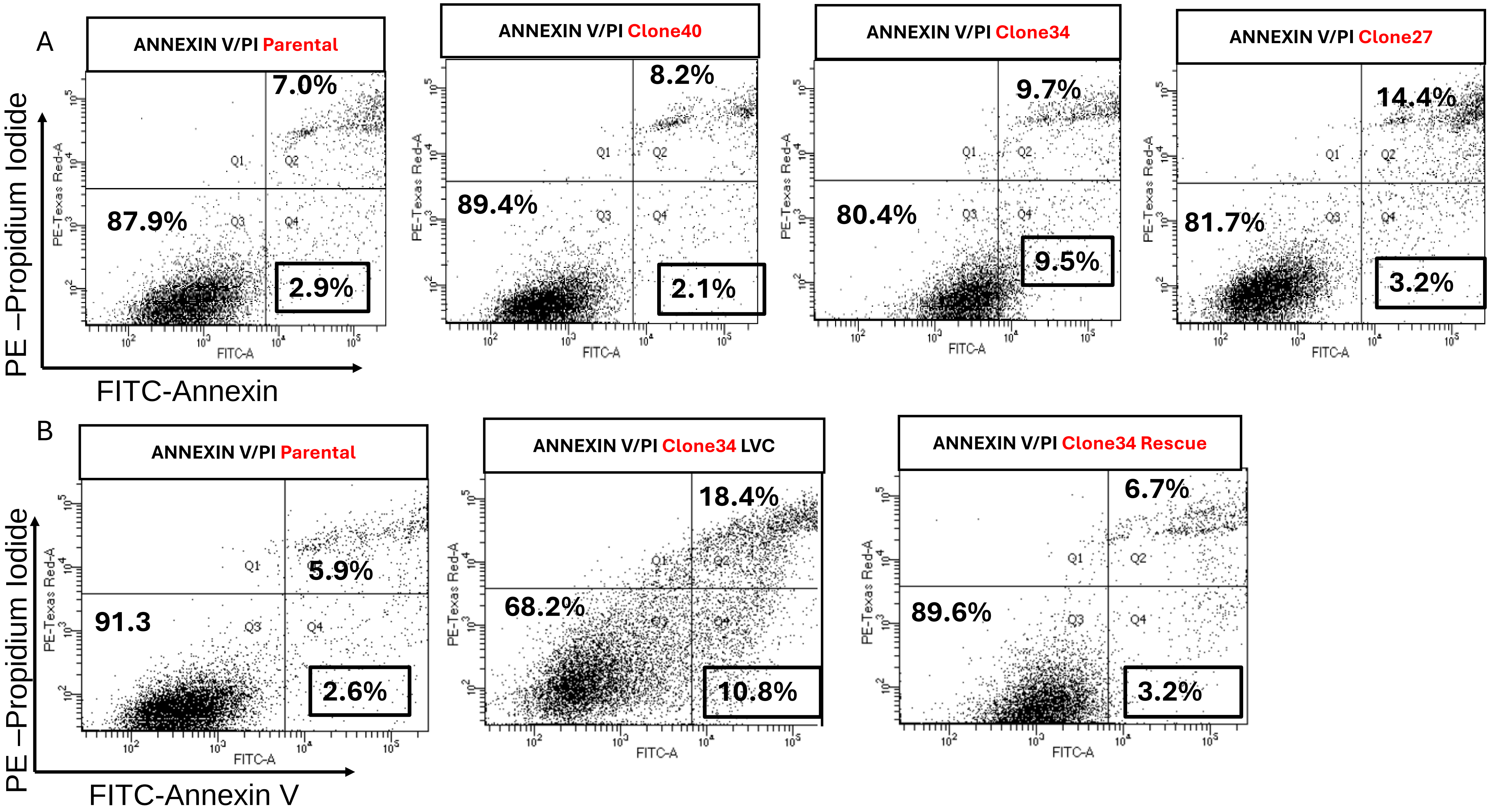
A
ANNEXIN V/PI Parental
7.0%
87.9%
2.9%
ANNEXIN V/PI Clone40
8.2%
89.4%
2.1%
ANNEXIN V/PI Clone34
9.7%
80.4%
9.5%
ANNEXIN V/PI Clone27
14.4%
81.7%
3.2%
PE –Propidium Iodide
FITC-Annexin
B
ANNEXIN V/PI Parental
5.9%
91.3
2.6%
ANNEXIN V/PI Clone34 LVC
18.4%
68.2%
10.8%
ANNEXIN V/PI Clone34 Rescue
6.7%
89.6%
3.2%
PE –Propidium Iodide
FITC-Annexin V
PE-Texas Red-A
FITC-A
Q1
Q2
Q3
Q4

Figure S5. Knockout of ALDH1A1 increases apoptosis. (A) Representative images of flow cytometry analysis of Annexin V -positive cells in ALDH1A1 knockout clonal cell lines compared to the parent. Boxed number represents Annexin V positive cells. **(B)** Apoptosis analysis by Annexin V/PI analysis in the rescue cell line.
FITC= Fluorescein isothiocyanate, PI = propidium iodide, PE= Phycoerythrin

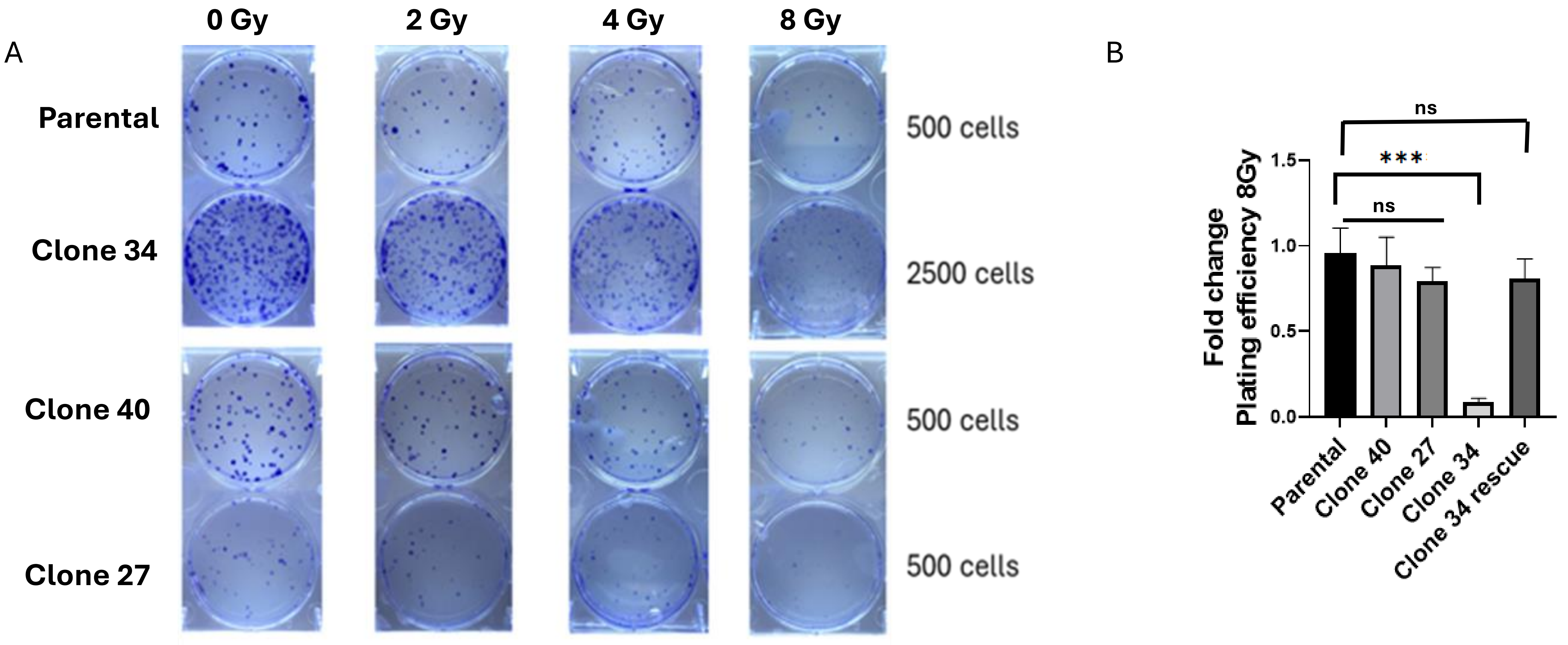
A
0 Gy
2 Gy
4 Gy
8 Gy
Parental
Clone 34
Clone 40
Clone 27
500 cells
2500 cells
500 cells
500 cells
B
Fold change
Plating efficiency 8Gy
0.0
0.5
1.0
1.5
ns
***
ns
Parental
Clone 40
Clone 27
Clone 34
Clone 34 rescue

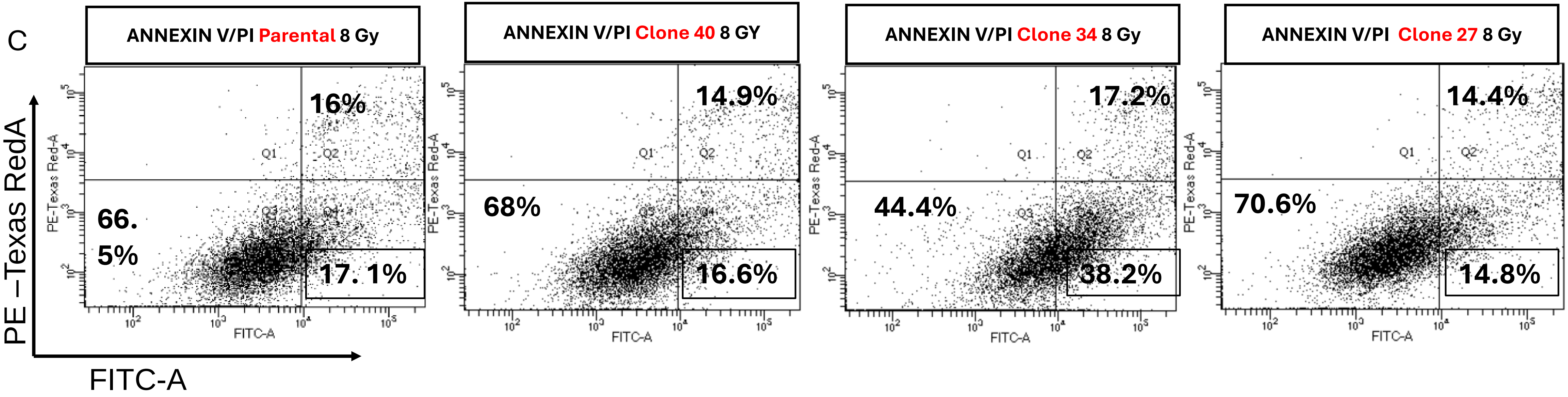
C
ANNEXIN V/PI Parental 8 Gy
ANNEXIN V/PI Clone 40 8 GY
ANNEXIN V/PI Clone 34 8 Gy
ANNEXIN V/PI Clone 27 8 Gy
16%
66.
5%
17. 1%
14.9%
68%
16.6%
17.2%
44.4%
38.2%
14.4%
70.6%
14.8%
Q1
Q2
Q3
Q4
PE-Texas Red-A
FITC-A
PE –Texas RedA
FITC-A

Figure S6. Colony formation in ALDH1A1 knockout clones. (A) Representative images of colony forming assay, comparing different radiation doses. **(B)** Fold change in colony forming efficiency in ALDH1A1 parental and knockout clones after exposure to 8 Gy radiation represented as fold change compared to the parental cell line. The loss of colony forming efficiency in clone 34 could be rescued by overexpression of ALDH1A1. **(C)** Apoptosis after exposure to radiation at 8 Gy. Box represents Annexin V positive cells. Data represents the mean ± standard deviation normalized to respective parental cells. ***$p < 0.001$, ns= not significant FITC= Fluorescein isothiocyanate, PI = propidium iodide, PE= Phycoerythrin

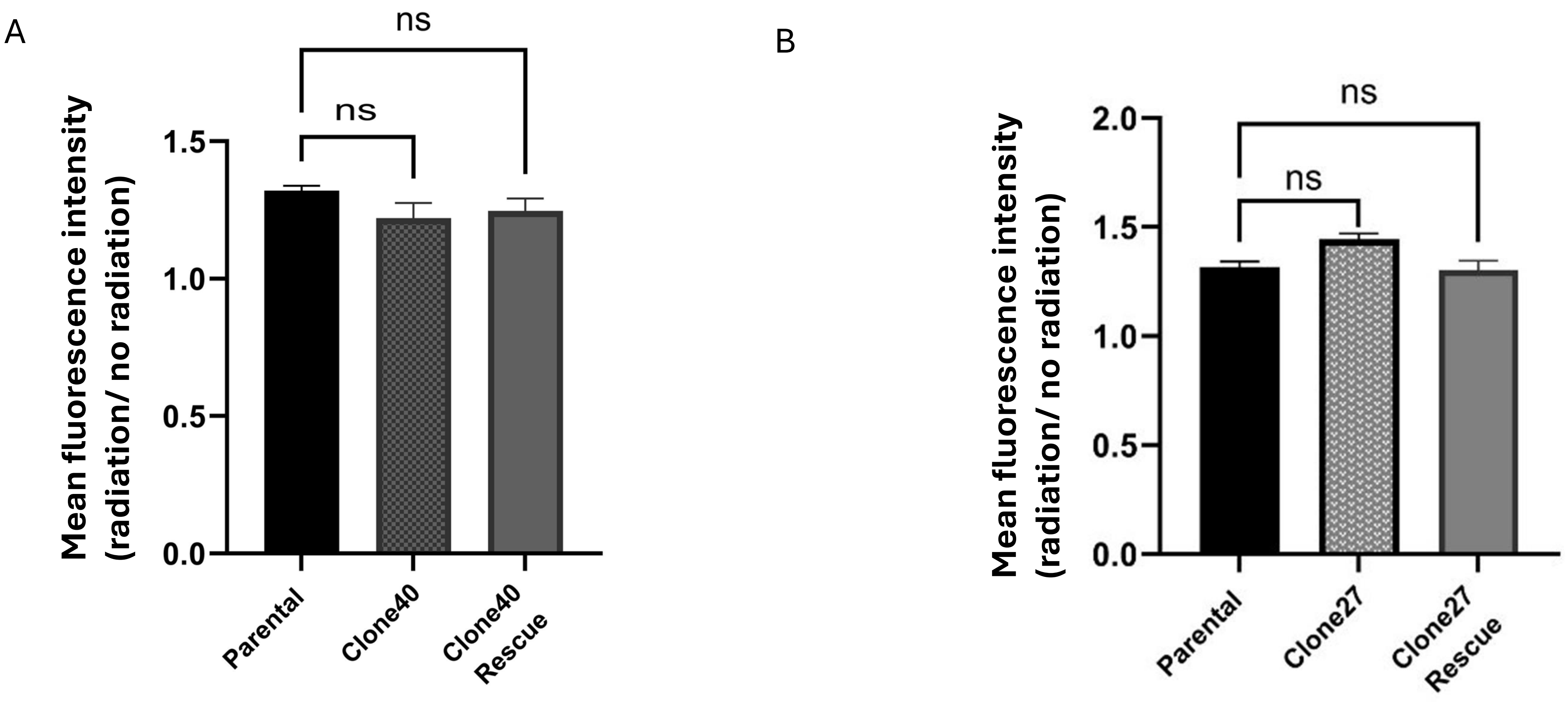
A
ns
ns
Mean fluorescence intensity
(radiation/ no radiation)
1.5
1.0
0.5
0.0
Parental
Clone40
Clone40
Rescue
B
ns
ns
Mean fluorescence intensity
(radiation/ no radiation)
2.0
1.5
1.0
0.5
0.0
Parental
Clone27
Clone27
Rescue

Figure S7. Loss of ALDH1A1 does not significantly affect reactive oxygen species levels in clones 40 and 27 after treatment with 8Gy radiation. Reactive oxygen species as measured as mean fluoresence intensity represented as radiation over no radiation in clone 40 **(A)** and 27 **(B)**, respectively. Data represents the mean ± SEM normalized to respective parental cells. ns = not significant.
